# Countertransference, alliance, and outcome in the treatment of patients with personality disorder: a longitudinal naturalistic study

**DOI:** 10.3389/fpsyt.2024.1490056

**Published:** 2024-12-24

**Authors:** Randi Breivik Øvstebø, Geir Pedersen, Theresa Wilberg, Jan Ivar Røssberg, Hanne-Sofie Johnsen Dahl, Elfrida Hartveit Kvarstein

**Affiliations:** ^1^ Section for Treatment and Research, Department of Research and Innovation, Division of Mental Health and Addiction, Oslo University Hospital, Oslo, Norway; ^2^ Institute of Clinical Medicine, University of Oslo, Oslo, Norway; ^3^ Department of Psychology, University of Oslo, Oslo, Norway

**Keywords:** countertransference, personality disorders, alliance, Feeling Word Checklist, non-completion

## Abstract

**Objective:**

Relational dynamics, including countertransference responses and the therapeutic alliance, are crucial in the treatment of patients with personality disorders (PD). However, few studies on PD treatment focus on the dyadic process of therapy. The present study aims to investigate associations between therapist emotional response/countertransference (CT) and patients’ experience of treatment alliance, and CT developments in therapies with treatment completion as outcome.

**Method:**

A longitudinal, observational study of patients (*N* = 365) treated at PD treatment units within specialist mental health services. CT was assessed repeatedly during therapy by the Feeling Word Checklist – Brief Version with three subscales—*Inadequate*, *Confident*, and *Idealized*. Early alliance was assessed after 6 months of treatment (Working Alliance Inventory, WAI). Treatment completion was defined as completing treatment according to schedule versus not completing treatment. Statistical analyses included Linear Mixed Models.

**Results:**

In the early phase of therapy, lower WAI predicted lower levels of *Confident*, *Idealized*, and higher *Inadequate* CT. The relation between early WAI and CT levels during treatment remained stable. The development of CT during treatment differed according to treatment completion with significant trends of increasing *Inadequate* CT and decreasing *Idealized CT* in not completed treatments. WAI and treatment completion had strong and independent effects. Further moderator analysis did not yield additional information.

**Conclusion:**

The study demonstrates significant associations between negative CTs and lower patient-rated WAI in the early phase of therapy, and a development of increasingly more negative CTs during therapy in treatments which were not completed according to schedule. The results indicate high clinical relevance of monitoring therapeutic relationships in PD treatments. Further research on the emotional and relational quality of psychotherapeutic relationships in PD treatments is needed.

## Introduction

Personality disorders (PDs) are prevalent disorders (1, 2), with a reported occurrence of 50% in psychiatric outpatient settings (3). Several studies have shown that psychotherapy is an effective treatment for patients with PDs (4–6). However, this patient group is known to evoke particularly challenging emotional responses in therapists (7–12), which makes the treatment more demanding and at greater risk of failing (13). The presence of PD has been shown to have a negative effect on treatment outcome (3) and early treatment termination is more frequent in this group (14). Factors predicting dropout have been found to include commitment to change, impulsivity, and the therapeutic relationship (15, 16). Two recent studies on therapists’ emotional responses (countertransference) when working with patients with PDs indicated that it is not the severity of the patient’s symptoms that appears to be most decisive in their relationship with the therapist. Instead, the patients’ relational problems and personality pathology had a significant impact on the therapist’s emotional response (7, 17). Patients with PD have deep-rooted interpersonal difficulties and tend to unconsciously project more intense and difficult feelings onto their therapists. This can significantly impact the therapeutic alliance. Focus on the relational dynamics is especially important in the treatment of patients with personality disorder (13). However, systematic research on these aspects is insufficient (3).

Therapist emotional response/countertransference (CT) is extensively described in clinical and theoretical literature since Sigmund Freud introduced the concept in 1910 (18). The prevailing view is that countertransference responses involve a joint creation with contributions from both patient and therapist, even though there is an ongoing discussion about the relative contribution from both participants. Although the concept originally derives from psychoanalytic theory, it is now considered an important aspect of the therapeutic process in diverse forms of therapy (9, 19). There are several definitions of CT (20), and it varies whether CT is measured by self-report or observer-ratings. In the present study, we assessed the therapists’ self-reported affective CT response to the patient. This is considered one part of the total CT construct, i.e., the feelings which therapists become aware of, acknowledge, remember, and are willing to report after a session (21).

Empirical research on CT and PD has been limited, but this is changing with increasing number of studies in recent years. To summarize, a consistent finding is that specific PD patterns/diagnoses tend to elicit specific countertransference responses across psychotherapists, suggesting that therapists overall reactions toward patients may be a source of valuable diagnostic information (8, 22, 23). For example, narcissistic PD is associated with angry/criticized and helpless/disengaged CT (24, 25) and borderline PD is e.g. associated with special/idealized (8, 26) and inadequate CT (7, 11, 23) Another consistent finding is that patients with more severe personality pathology is associated with more negative CT (7, 27) and patients at the DSM-IV-TR (APA, 2000) cluster A and B is associated with more negative and varied CT responses than those with cluster C (8, 28).

One of the most consistent findings in psychotherapy research is that the therapeutic alliance (TA; the mutual collaboration and partnership) between therapist and patient is a key predictor of treatment outcomes (29–32). Both patient and therapist-rated alliance have been found predictive (32–37). The capacity to *form* an alliance is assumed to be a quality that both the patient (38) and the therapist (39) brings to treatment (29). Research suggests that the therapist play a significant role (40), but patients undoubtedly also contribute to the therapeutic collaboration that arises between them (29). Emotional processes are found to play an important role in his respect (41, 42). Several studies have emphasized the importance of the therapists’ ability to recognize and manage their emotional responses for the development of a productive alignment between patients and therapists (therapeutic alliance) - a process where treatment can be completed (20, 43).

Due to their substantial difficulties in interpersonal relationships, patients with PDs face particularly significant challenges in forming a therapeutic alliance (44, 45). Studies have shown that therapists find it especially challenging to maintain a good alliance with cluster B patients (25, 29, 45). A recent study indicated weaker early alliance among patients with borderline PD (BPD) (46). While a strong positive therapeutic alliance is predictive of more successful treatment outcomes, strains and ruptures (episodes of tension or breakdown, that is, countertransference and transference enactments (47)) in the alliance can result in premature termination of treatment (44). According to Hayes et al. (20), acting out of CT is typically harmful, though not necessarily irreparable. Several studies support the view that ruptures in the alliance are unavoidable, and when negotiated, they can present opportunities for therapeutic change (43, 48). However, when the therapist is under great pressure in the moment, the anxiety level increases and can evoke strong feelings of inadequacy and negativity in the therapist. Studies have shown that therapists often react by avoiding these feelings and resorting to maladaptive strategies to manage them (49, 50).

Despite the importance of the relational dynamics (including both TA and CT) in treating patients with PD, few studies have investigated this association. These vary in respect to the operationalization of TA and whether TA is patient-rated and therapist-rated. Most of the studies are correlational in nature and all studies have found meaningful correlations between alliance and different aspects of CT responses (21, 26, 39, 51–55). Only two of these examined alliance and CT in a PD population specifically. One of these, based on the same participants as the current study sample, found that patient rated alliance showed a negative correlation with *Inadequate* CT and positive correlation with *Confident* CT (26). Tanzilli et al. (25), investigating therapists emotional response and therapeutic alliance when treating adolescent patients with narcissistic personality subtypes found that lower quality of therapeutic alliance was associated with the grandiose narcissistic subtype (strongly related to the personality syndrome defined in DSM-IV) which was also correlated to angry/criticized and disengaged/hopeless response. Two recent studies have also examined CT and alliance in more detail during the psychotherapy process (43, 56), however, not in a PD population. In their sample of depressed patients, Falkenström and Holmqvist (56) found poorer working alliance and less engagement among patients where the therapist experienced negative feelings in sessions. Therapists’ positive feelings related to a better working alliance, patients feeling more engaged, and therapists’ positive feelings also had a positive effect on outcome. Tishby and Wiseman (43), in their sample of students with mostly mild to moderate depression, and difficulties in relationships, found that therapist negative CT patterns were associated with more alliance ruptures and less rupture resolution with patients. The results indicate that therapist’s own unresolved conflicts may impact countertransference negatively, and the authors suggest that increased countertransference awareness helps therapists recognize and address ruptures, initiating resolution processes (43, 57).

In meta-analyses, early alliance has been classified as first to fifth session, except from Frank and Gunderson’s long-term study (58, 59). In their study, alliance measures taken in the 6^th^ month of treatment were considered “early”. The present study also employs a 6-month measure, as many of the therapies in the present study are long-term, lasting up to 60 months at the most. As far as we know, no previous study has investigated patient-rated early alliance as a predictor of therapist CT longitudinally in therapies with PD patients.

Early treatment termination is more frequent among PD patients and represents a prevalent issue in clinical practice. The reasons for non-completion in psychotherapy are multifaceted and include situational, personal and relational factors, including the therapeutic relationship (50). More studies are needed to investigate what happens in the interactions with PD patients who do not complete therapy. Particularly relevant is therapists’ countertransference (CT) during challenging therapies. In the present study, completion of psychotherapy according to scheduled plan was the chosen outcome. To the best of our knowledge, only one study has examined CT and treatment course in a sample of patients with PD specifically. Røssberg and co-workers (28) found strong correlations between CT feelings and clinical change during treatment. Patients who dropped out of treatment evoked significantly more negative CT reactions in the initial treatment phase than patients who completed treatment.

### Aims of the present study

There is still a scarcity of studies examining the treatment processes in therapies for patients with PDs. A recent former study from the same data collection emphasized the positive potentials of PD treatment units implemented within specialist health services by demonstrating how therapists’ feelings of inadequacy were generally low and stable over time, and therapists’ feelings of confidence increased over time (7). The present study is a further longitudinal investigation of countertransference responses (CT) among therapists in treatment units treating patients with PD and personality-related problems. It explores the relation between therapist CT and patient-rated early alliance (WAI) and includes investigation of treatment completion, the latter an issue of high relevance in clinical practice. The study aimed to answer the following research questions:

1. Is alliance in an early stage of therapy (after 6 months) a predictor of therapist CT development during treatment?
2. How do therapists’ CT responses develop in therapies with different clinical outcome (treatment completers versus non-completers)?

## Materials and methods

### Design

The present study is a naturalistic study with a longitudinal, multi-site, design.

### Setting

Data was retrieved from the quality register of the Norwegian Network for Personality Disorders (60) – a clinical research collaboration between outpatient units on a specialist mental health service level. The present study included data from the period 2010-2016 which involved 16 different outpatient clinics. The primary target group of the units is people with PDs and personality-related difficulties. The research setting is elaborated in a former publication (7).

### Participants

#### Patients

The study sample (N=365) included patients who had 1) terminated treatment, 2) were evaluated diagnostically with semi-structured interviews before treatment, 3) had at least one therapist rating of CT, 4) had completed patient rated alliance (WAI) at 6 months, and 5) had therapist-rated data on treatment completion. The 6-month evaluation is performed 6 months after the first evaluation on referral. The time in treatment at this evaluation point is 3-6 months, depending on the duration of the assessment phase and waiting list for the treatment programs.

#### Therapists

Therapist teams in the Network for Personality Disorders are elaborated in a former publication (7). The units are multidisciplinary collaborations including psychiatrists, psychologists, psychiatric nurses and social workers. The Network regularly provides updated courses and conferences on PD assessment procedures and therapeutic principles. Clinical discussions and supervision of treatment processes and CT responses are traditionally important elements of the treatment programs. The quality registry did not include therapist data systematically coupled with patient data. However, on the basis of an informal query among the treatment units, some general information was available: The mean number of therapists at each unit in the present study period was estimated to 9 (range 4 -18), approximately 75% of the therapists were female and mean age was 45 years. Mean length of clinical experience was 17 years and 73% of the therapists were formally trained (for 3 to 5 years) in group psychotherapy (61, 62). It was mainly the patients’ individual therapist who filled out the FWC-BV.

### Treatment

The applied treatments are elaborated in a former publication (7). The different outpatient units combined psychoeducational, group and individual psychotherapy formats. Treatment approaches were mainly psychodynamic, but combinations also included body awareness, art and cognitive therapies. Specific PD approaches implemented within some units in the Network during the investigation period include mentalization-based therapy, dialectical behavioral therapy and schema-focused therapy. At each unit, the allocation of patients to therapists was random in the sense that after the initial assessment, patients had to wait for the next available therapist. Mean duration of PD treatment was 20.3 months (SD 10.6). 69% were treatment completers (completed therapy according to plan) while 31% were non-completers (dropped out or ended therapy of different reasons, e.g. advised to end treatment, referred to other treatment, moved out of region, or for other reasons).

**Diagnostic assessment**. All patients were diagnosed according to the DSM-IV (63) by use of the Structured Clinical Interview for DSM-IV Axis II (SCID-II) for PD (64), and the Mini International Neuropsychiatric Interview (MINI) (65) for symptom disorders. Diagnostic reliability was not investigated. However, diagnostic assessments were performed in each unit by clinical staff who had received systematic training in diagnostic interviews and principles of the Longitudinal, Expert, All-Data (LEAD) procedure (66, 67). Diagnostic assessment was performed at baseline (when patients were referred to treatment).

### Ethics

The quality register of the Network includes anonymized clinical data transferred from each treatment unit to the register database. The collection of anonymous, clinical data to the quality register requires informed, written consent from all participating patients. Data collection procedures at each contributing unit are approved by local Data Protection Officers. Data security procedures for the quality register are approved by the data protection officer at the responsible center for the research (Oslo University Hospital). Since the data in the quality register are anonymous, formal approvals from the Norwegian State Data Inspectorate and Regional Committee for Medical Research and Ethics are not required.

### Measures

**Feeling Word Checklist-BV**. To assess the therapists’ countertransference, we used the Feeling Word Checklist-BV (FWC-BV) (26). FWC-BV is a brief version of the FWC-58 (68), comprising 10 feeling words (26). The therapists were asked to rate how strongly they experienced each item on a 5-point rating scale, ranging from 0 (not at all) to 4 (very much). Different versions of the FWC have been developed, varying in number of items (from 10-58). For an overview of FWC questionnaires, see e.g. Lindquist and colleagues (69). The items of the FWC-BV have shown to be reliably differentiated as three distinct dimensions *Confident, Inadequate and Idealized* (26). In the present study, therapist CT was assessed repeatedly during therapy, first every 3 months up to 12 months, then every sixth month until 60 months. In addition, CT was assessed at the end of therapy.

**Patient alliance (WAI-SR).** The patients filled in the WAI-SR (70, 71) every 6 months during treatment and at discharge from treatment. The WAI-SR is a 12-item questionnaire representing 3 different aspects of the patient’s relationship to the therapist: bond, task and goal. Patients are asked to judge each question on a Likert scale from ‘Never’ (1) to ‘Always’ (7). The patients filled out two versions of the WAI-SR: one with reference to their group therapist (WAI-G) and one with reference to their individual therapist (WAI-I). In the present study, only the WAI-I was analyzed.

**Assessment of treatment completion**. Treatment completion was assessed by the therapist when the patient ended therapy. Treatment completers included patients who completed therapy according to plan. Non-completers included patients who dropped out of therapy or ended therapy for different reasons (advised to end treatment, referred to other treatment, moved out of region, or for other reasons).

### Statistics

#### Longitudinal analyses

We used linear mixed model to analyze longitudinal data (mixed models, SPSS, version 27) (72). The dependent variables were each of the three FWC-BV subscales (*Inadequate, Confident* and *Idealized*) (26). All analyses were performed on the total sample of 365 patients.

Analyses started with three separate open models for each CT variable. Adding linear time (months from baseline) improved model fit (reduction in log likelihood estimations) in all three models. The final model for further analyses included a linear structure with random intercept and slope (critical values for chi-square statistic: *p<*0.01) and an unstructured covariance type. In order to restrict linear inflation of scores, we added all the CT scores registered at termination to the corresponding last time point in every longitudinal patient course. The main analyses investigated longitudinal change in the three CT subscales and variation associated with WAI and patient treatment completion. As described in a former study, there was a general trend of no significant clustering of CT change patterns within specific treatment units (7).

We investigated each of the WAI subscales (Bond, Task and Goal) and the total WAI score (WAI_total_) as separate predictors in each of the three CT models. Similarly, we investigated the dichotomous variable; patient treatment completion and CT development (**Table 1**). Lastly, we investigated interactions between CT development, early WAI and treatment completion. The model included predictor interactions for both completer and WAI and the moderator interactions, completer * early WAI and completer * early WAI * time.

**Table 1:** Therapist countertransference over time in subgroups with different clinical outcomes and early WAI as predictor.

Model	Predictor	Intercept	p	Slope	p	Explained intercept variation	Explained slope variation	AIC
		Mean (SE)		Mean (SE)		%	%	
INADEQUATE		0.42 (0.03)	**<.001**	0.005 (0.002)	**.038**	Reference*	Reference*	1410
	WAI	-0.18 (0.03)	**<.001**	0.0007 (0.002)	.724	19	0	1345
	Non-completer vs completer	-0.15 (0.07)	**.038**	0.03 (0.005)	**<.001**	0	17	1356
CONFIDENT		2.71(0.05)	**<.001**	0.006(0.002)	**.016**	Reference*	Reference*	2088
	WAI	0.16(0.04)	**<.001**	0.0002(0.002)	.926	8	1	2064
	Non-completer vs completer	-0.06(0.10)	.561	-0.01(0.005)	**.049**	0	0	2081
IDEALIZED		1.05(0.05)	**<.001**	0.008(0.003)	**.003**	Reference*	Reference*	2231
	WAI	0.13(0.045)	**.004**	0.0005(0.003)	.854	5	0	2220
	Non-completer vs completer	0.09(0.11)	.394	-0.03(0.006)	**<.001**	1	14	2213

LMM estimations with baseline levels (intercept) and monthly change-rate (slope estimates) for 3 dependent variables (CT subscales: INADEQUATE, CONFIDENT and IDEALIZED). The table presents estimated deviance of intercept and slope and explained variance associated with subgroups with different clinical outcome, and estimated deviance of intercept and slope and explained variance associated with early alliance for the three subscales. Indicator of model fit is Akaikes Information Criterion (AIC), where smaller is better. The reference values for calculating explained variance is the variance estimates in the initial open model for each dependent variable. A significant variation estimate in the initial model (p<0.05) is given by Reference *. Bold = *p*-value is significant at the 0.01 or 0.05 levels.

### Unbalanced data

Missing data in the current study sample were firstly, due to different locally occurring, administrative failures of delivery or registration to the quality register (60). Secondly, the patients had different treatment duration which caused natural differences in number of CT assessments. Mean number of FWC-BV assessments per patient was 3.1 (SD=1.5, range 1-9), 9% had one CT assessment, 34% had two assessments and 60% had three or more assessments. To investigate possible systematic bias of missing longitudinal assessment points, we investigated a variable counting the number of assessment points as a longitudinal predictor in separate models for the three dependent variables (73). Number of assessments was not associated with deviating longitudinal CT levels of all three CT dimensions. In a final model, we analyzed the impact of treatment duration and treatment completion on therapist CT. Treatment duration was not associated with deviating longitudinal CT levels of *Inadequate*, *Confident* and *Idealized*.

## Results

### Descriptive data

Mean age of patients was 33 years (SD=10) and 77% were females. The majority (92%) fulfilled criteria for one or more Axis I diagnosis of the DSM-IV, of which mood and anxiety disorders were most frequent. Approximately 75% fulfilled the criteria for one or more personality disorders (PD). Mean number of fulfilled SCID-II criteria was 10.0 (SD 6.7). Borderline PD (26%), Avoidant PD (35%) and PD NOS (17%) were the most frequent PD diagnoses. The mean Global Assessment of Functioning score (GAF; APA, 1994) was 49.6 (SD: 5.9), a score within the “Severe” range according to APA. Overall, patients rated high initial levels of working alliance (Mean 5.36, SD 1.08). Among the treatment completers (n=250, 69%) average treatment duration was 22 months (SD 11.3), and among non-completers (n=115, 31%) 16.6 months (SD 7.8). Differences in early WAI between completers and non-completers were small, but significant (completers: Mean 5.45, (SD: 1.02), non-completers 5.19 (SD: 1.18) (*p*<0.05, independent samples *T*-test). Descriptive data are given in **Table 2**. Differences between the two outcome subgroups (**Table 2**) were minor (*p*>0.1, independent samples T-test), with some noteworthy distinctions. In the non-completer group, significantly more patients had comorbid Paranoid PD. There were also significantly more patients with OCD, PTSD, substance disorder and more patients were not living in a close relationship and had no months of work/study previous year in the non-completer group.

**Table 2:** Baseline characteristics in subgroups: completers vs non-completers.

	Completers N=250			Non -completers N=115		
	Mean	SD	%	Mean	SD	%
			69			31
Age	33.5	10.3		31.8	9.9	
Female			74			84*
No work/study last year			39			55*
Not living in a close relationship			56			63
Axis I diagnosis						
Major depression			53			46
OCD			1			7*
Panic disorder			16			9
Agoraphopiba			3			6
Social fobia			20			22
Generalized anxiety disorder			11			6
Somatization			2			1
PTSD			11			20*
Eating disorder			17			13
ADHD			4			1*
Mood disorder			64			57
Anxiety disorder			47			50
Substance disorder			2			7*
Total number symptom disorders	1.59	1.0		1.6	1.1	
Axis II diagnosis						
Paranoid			3			11*
Schizoid			0			0
Schizotypal			0			0
Antisocial			1			4
Borderline			23			32
Histrionic			0			0
Narcissistic			1			0
Avoidant			33			38
Dependent			5			4
Obsessive-compulsive			3			4
PD NOS			15			18
No PD			28			20
Number of PD criteria	9.5	6.7		11.1*	6.4	
More than one personality disorder			13			17*
Self-harming last 12 months			58			67
Suicide attempt last 12 months			12			14

Descriptive data with mean values, standard deviations (SD), and valid percent (%). Completer= completed according to plan. Non-completer= drop-out or ending therapy of different reasons.

Significant differences are marked with * (*p*<0.05, Person chi-square test/independent samples *T*-test).

### Longitudinal analyses

The LMM analyses were conducted on the total study sample (**Table 1**). Overall, the CT levels increased slightly over time (*Confident, Inadequate* and *Idealized*). Levels of *Inadequate* CT were lowest and *Confident* highest.

### Variation associated with early WAI

Higher early WAI_total_ predicted significantly higher initial *Confident* and less initial *Inadequate* CT levels. Initial levels of WAI_total_ accounted for 19% of the initial *Inadequate* variation, 8% of the initial *Confident* variation and 5% of the initial *Idealized* variation. For all three CT responses, differences in early WAI_total_ levels were not associated with differences in CT change over time (**Table 1**). Investigation of the three WAI subscales (bond, task and goal) revealed corresponding trends as WAI_total_ for all three subscales.

### Variation associated with treatment completion

**Figure 1:**
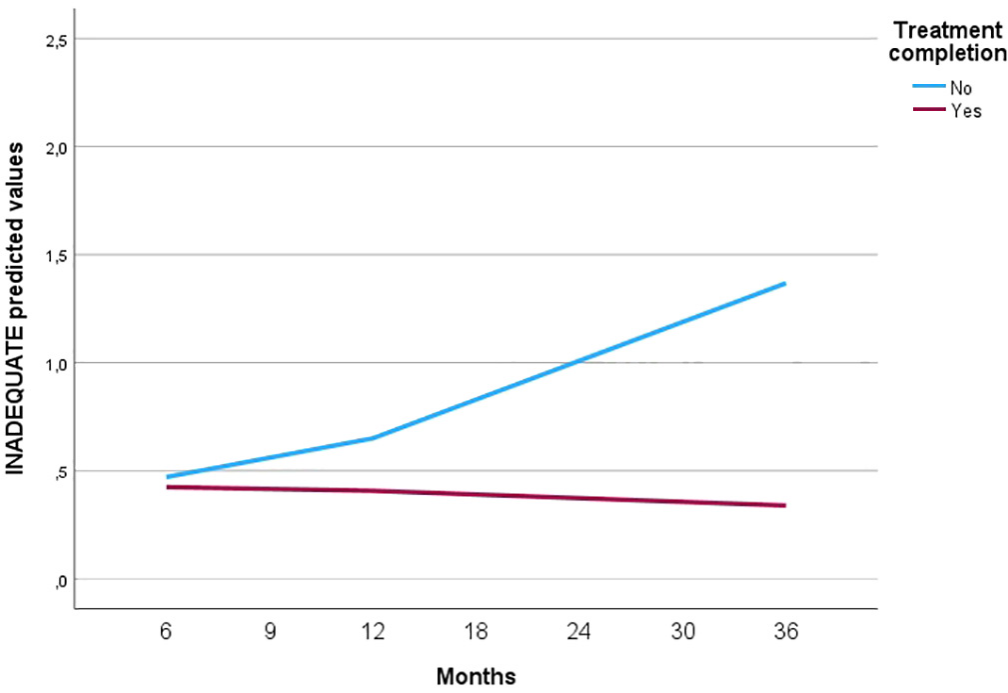
Countertransference development in subgroups with different clinical outcomes.

Initial levels of CT did not differ by subgroup (treatment completers and non-completers) but change over time was significantly different by subgroup in the *Inadequate* and *Idealized* CT dimensions (*p*<0.001) (**Table 1**, **Figure 1**). For *Confident*, there was only a borderline significant trend (*p*=0.049). Treatment completion accounted for 17% of the *Inadequate* slope variation and 14% of the *Idealized* slope variation, but no further variation of *Confident* (0%).

### Interactions treatment completion * early WAI

Further investigation of interactions between CT development, early WAI and treatment completion, revealed no significantly distinguishing moderator effects (*Inadequate* CT *p*_intercept_ = 0.855, *p*_slope_ = 0.456, *Idealized* CT *p*_intercept_ = 0.432, *p*_slope_ = 0.338, *Confident* CT *p*_intercept_ = 0.266, *p*_slope_ = 0.707. Previously demonstrated predictor effects were maintained in this model including both predictors.

## Discussion

The present study explores the essential therapeutic dyad in treatment of patients with considerable emotional, relational and attachment issues. As a naturalistic, clinical study, the sample is large, and representative of PD-focused treatments as applied within specialist mental health services in Norway.

The main findings are the following: Firstly, the demonstration of significant, independent associations between early negative CTs and lower early patient rated alliance. More specifically, lower ratings of WAI were associated with lower therapist rated *Confident and Idealized* CT and higher initial *Inadequate* CT, and conversely, higher early WAI with significantly higher initial *Confident and Idealized* and less initial *Inadequate* CT levels. It thus seems that the quality of early WAI as experienced by patients was largely in concordance with the quality of therapists’ experiences in the same phase of treatment, which is in line with other correlational studies on CT and alliance (21, 26). Moreover, the study demonstrated that the relation between early alliance level and CT response levels remained stable throughout therapy. That is, high or low levels of early alliance did not predict any deviating pattern of CT development during therapy. If alliance was low at the start of treatment, our models indicated that positive CT responses would start from a lower level, follow the general change trend in the sample and increase over time, but this lower level would still prevail throughout treatment. Our findings underline the importance of the quality of the starting point in therapy. Investigation of parallel developments of alliance and CT during the therapy was outside the scope of the present study but could further illuminate qualities of the change process in PD treatment.

It should be mentioned that overall, patient-measured alliance was high. Although early alliance had a highly significant impact on CT, the actual change in intensity on the CT scale was low. Possible explanations for the predictor-associated small nuances in CT intensity are the same as those described in a previous publication (7). That is, relatively infrequent measurement points (e.g. no immediate reactions after sessions) very likely contribute to less intense emotional responses on the CT scale. Nevertheless, the levels are comparable to those reported in other studies that have examined CT, using various versions of FWC as instrument. The significant findings in the present study are interesting because they show two non-complementary perspectives, namely the patient and the therapist, and demonstrate the importance of CT and the interaction between the two participants in the therapeutic dyad.

The relatively high early alliance ratings might seem surprising. Patients with PDs are known to struggle with significant impairment in interpersonal relationships and there are often problems in the formation of a therapeutic alliance (44, 46). However, several other studies examining patients with severe relational problems have reported alliance (WAI) within the same range (74, 75). In a study by Folmo and colleagues, BPD and alliance development were examined. The patients in their study are from the same quality register as the present study. They found WAI levels within the same range and the overall picture of change over time was a significant increase of all three working alliance subscales (75). There might be several reasons for the relatively high WAI scores in general. It might illustrate the potential value of PD treatment teams with experienced therapists in specialist health services. It is also natural to consider other explanations. Another study, using a different alliance measure (CALPAS) found that therapists’ alliance ratings were consistently lower than patients’ rating over time (45). This might also be the case in the present study; however, we do not have therapists’ assessments of alliance. Lastly, the phenomenon of self-serving bias is always present when using self-report as a method. That is, it is possible that the patient may not be able to be honest and may find it difficult to report negative feelings about the therapist. Research suggests that self-report instruments may be particularly problematic for patients with PDs (especially, narcissistic, paranoid, and antisocial), who often lack self-awareness and tend to defensively downplay or deny their psychopathology (25, 76). Our study has, however, not differentiated between different types of PDs in relation to alliance nor examined the longitudinal alliance development. To summarize, even though the overall alliances scores were generally high, lower early alliance was significantly related to more negative CT in therapists. This conveys the idea that the impact of early alliance continues or remains relevant for the therapeutic relationship during the course of therapy. As to our knowledge, no other study have investigated this association.

The second main finding is the demonstration of significant associations between the developments of increasingly negative CT responses in therapists during treatment with non-completers as compared to completers. The negative CT development in non-completed treatments was irrespective of the quality of early alliance. In the previous study on CT from the same quality register (7) we found that, overall, patients who did not improve were associated with more negative CT. The previous study did not investigate treatment completion as an aspect of outcome. Nonetheless, meeting to therapy and completing according to the scheduled plan, is likely related to the treatment process itself. Several studies suggest that patients with more PD pathology elicit more negative feelings in therapists (7, 12, 21, 22) and clinical studies report high drop-out rates for PD patients in general (77, 78) and BPD in particular (79). Our study has not delved into further investigations of mediating or moderating variables of non-completed therapies, such as the type of PD, severity of PD, or other comorbid conditions as mentioned above. This would be beyond the scope of this study, but will be important aspects to investigate in future studies. In contrast to Røssberg and coworkers study (28), which found that dropouts very early in therapy were associated with more negative CT, the present study shows a more consistent early baseline regarding CT levels. Differences emerged more over time, with increasing negative CT in the non-completer group. This may indicate that something happens in the process along the way, which is not necessarily captured at the start of therapy. It should be noted that the present study and Røssberg and coworkers study are different in several ways, and thus not directly comparable, e.g. “early” in our study refer to WAI at 6 months. Further, the study designs are different, with correlational design versus longitudinal analyses in the present study. In addition, their study examined drop-outs, while the present study examines a somewhat wider definition of non-completer (dropped out or ended therapy of different reasons).

The therapists in this study were generally experienced, working in specialized units, where CT is part of their daily routine and systematic supervision is recommended. Despite their experience, the study shows they are negatively affected over time by patients who do not complete treatment as planned. However, we do not know whether negative CT *predicts* non-completion. This is an interesting question that should be addressed in futures studies. Further studies of the psychotherapy process are needed, including mutual alliance, and effects of therapist’s supervision and training on negative CT. However, this quality register with few assessments over time did not allow for such detailed investigations. It could be mentioned that there is an increasing research interest in examining treatment non-completers and the impact of the therapeutic relationship, with ongoing research in this area (50).

Comparing the non-completer group to completers, the former was generally characterized by significantly greater PD comorbidity and overrepresentation of paranoid PD. This may not be surprising, as the specific features of paranoid PD are likely to be of significance for the formation of a trusting therapeutic relationship, thus potentially complicating the process of therapy. Comorbid paranoid PD features, are, however, quite common in more severe PD conditions presenting to health services. The non-completer group was also characterized by more individuals with PTSD, OCD, eating disorders and substance disorder, overall indicating that the non-completer group represented a poorer functioning patient group. This illustrates the important clinical challenge of such severe conditions. A recent study of severely self-harming PD patients in repetitive, inpatient situations, were indeed characterized by such complex comorbidity (80). Interestingly, as not correspondingly reflected in therapist ratings of CT, the non-completer group and the completer group differed on patient-reported alliance with significantly lower early alliance ratings in the non-completer group. However, as mentioned, the mean scores were relatively high, and score levels in both groups were within a satisfactory range. Thus, inferences from WAI scores alone may not be sensitive enough in identifying patients with risk of non-completion. The discrepancies between patient-rated alliance and therapist-rated CT underline that a more fine-tuned investigation of the starting point in therapy, patients’ vulnerability and therapists’ competence could reveal more nuances on how the process develops and the mutual understanding and bond in therapeutic dyad develops.

Patients with PDs still pose significant challenges to psychotherapists. Their repetitive interpersonal struggles are often actualized in the therapeutic relationship, hindering the formation of a positive alliance and evoking difficult emotional responses in therapists, which puts therapists’ performance under pressure. There is a growing body of research on strategies to enhance therapists’ abilities to navigate these pervasive challenges. In general, therapists are advised to engage in reflective practices that increase their self-awareness, affect regulation and interpersonal sensitivity (43, 57, 81). Meta-analytic evidence (20) suggest that therapists may prevent countertransference enactment and reduce non-completion if they attend regular supervision, develop emotional regulation strategies, such as mindfulness-based techniques, set healthy boundaries with their patients, adopt non-defensive stances and practice self-care, to name a few. Furthermore, therapists are encouraged to commit to ongoing personal therapy to address own, unresolved issues. Numerous training programs are currently being developed to enhance therapists’ abilities to tolerate intense emotions, including negative ones such as anxiety, anger, and stress e.g (82, 83).

In conclusion, the present study shed light on the underlying CT processes in PD treatments, emphasizing the important impact of relational factors in the bi-directional nature of the therapeutic relationship. Finally, it must be emphasized, that even though associations between CT, alliance, and treatment completion have been identified, we cannot make any claims about causality. The study highlights the need for further, longitudinal studies on the combined influence of therapist and patient factor in the therapeutic relationship. This may contribute to the development of more tailored interventions for individuals with personality disorders (50).

### Strengths and limitations

Clinical data are obtained from a quality register that includes real-life treatments and real patients struggling with PD and personality problems. There are few studies that have examined patient populations with poorly functioning PD patients. This makes the data highly clinically relevant. The weakness lies in missing assessments, partly due to varying treatment durations, which naturally occur in naturalistic, observational studies. However, we have conducted analyses to examine the impact of missing measurements and treatment duration, and these did not show significant bias.

Unfortunately, the data from the quality register do not allow for a more detailed study of the therapist qualifications, nor enable analyses of variation between therapists. Such analyses could, for example, have shown whether certain therapists were more inclined towards certain CT reactions.

A limitation with WAI used in the present study is that it typically measures the therapeutic alliance at a macro-level (84). More fine-grained measurements require a different study design than the present quality register allows, which has few, but longitudinal assessments over several months and years of treatment. Therefore, our study can only speak to overarching trends.

A weakness with self-reports (in this study patient rated WAI and therapist rated CT), is that both therapist and patient may underreport negative feelings due to lack of awareness of them or discomfort acknowledging negative responses. This might represent a bias. One way to address this problem in future studies is to use observer-based measures in addition. However, it is worth noting that the two self-reported measures in this study are clinically important as they are from two different perspectives, namely the patient and therapist.

## Conclusion

The study supports research indicating associations between a patient’s perception of alliance and therapist CT responses. Our study underlines the importance of the quality of the starting point in therapy. In addition, the study showed that non-completers were associated with increasingly negative therapist CT during treatment. This result was independent of variation in early alliance. This underscores the significance of continuous countertransference awareness when working with personality disorder patients. Further exploration revealed that comorbid paranoid PD, PTSD, OCD, and eating disorders were more frequent in the non-completer group. The study contributes to psychotherapy research emphasizing the value of the emotional and relational quality of psychotherapeutic relationships in the treatment of patients with PDs.

## Data Availability

Due to restrictions imposed by the Regional Medical Ethics Committee regarding patient confidentiality, data are available upon request. Requests for data may be sent to the hospital’s Privacy and Data Protection Officer at: personvern@ous-hf.no. Requests to access these datasets should be directed to personvern@ous-hf.no.
